# Exosome biogenesis: machinery, regulation, and therapeutic implications in cancer

**DOI:** 10.1186/s12943-022-01671-0

**Published:** 2022-11-01

**Authors:** Qing-Fang Han, Wen-Jia Li, Kai-Shun Hu, Jie Gao, Wen-Long Zhai, Jing-Hua Yang, Shui-Jun Zhang

**Affiliations:** 1 Department of Hepatobiliary and Pancreatic Surgery, The First Affiliated Hospital of Zhengzhou University, Zhengzhou, 450052 Henan China; 2 Henan Research Centre for Organ Transplantation, The First Affiliated Hospital of Zhengzhou University, Zhengzhou, 450052 Henan China; 3 Guangdong Provincial Key Laboratory of Malignant Tumor Epigenetics and Gene Regulation Medical Research Center, Sun Yat-Sen Memorial Hospital Sun Yat-Sen University, Guangzhou, 510120 China; 4 Henan Diagnosis & Treatment League for Hepatopathy, Zhengzhou, 450052 Henan China; 5 Clinical Systems Biology Key Laboratories of Henan, the First Affiliated Hospital of Zhengzhou University, Zhengzhou, 450052 Henan China; 6 Henan Engineering & Research Center for Diagnosis and Treatment of Hepatobiliary and Pancreatic Surgical Diseases, Zhengzhou, 450052 Henan China

**Keywords:** Exosomes, Biogenesis, Regulation, Implications, Cancer

## Abstract

Exosomes are well-known key mediators of intercellular communication and contribute to various physiological and pathological processes. Their biogenesis involves four key steps, including cargo sorting, MVB formation and maturation, transport of MVBs, and MVB fusion with the plasma membrane. Each process is modulated through the competition or coordination of multiple mechanisms, whereby diverse repertoires of molecular cargos are sorted into distinct subpopulations of exosomes, resulting in the high heterogeneity of exosomes. Intriguingly, cancer cells exploit various strategies, such as aberrant gene expression, posttranslational modifications, and altered signaling pathways, to regulate the biogenesis, composition, and eventually functions of exosomes to promote cancer progression. Therefore, exosome biogenesis-targeted therapy is being actively explored. In this review, we systematically summarize recent progress in understanding the machinery of exosome biogenesis and how it is regulated in the context of cancer. In particular, we highlight pharmacological targeting of exosome biogenesis as a promising cancer therapeutic strategy.

## Background

Exosomes are extracellular vesicles with a diameter of ~ 30–150 nm that are secreted by the fusion of multivesicular bodies (MVBs) with the plasma membrane [1]. Since they were first visualized and described in the 1980s [2, 3], especially in the last 10 years, the field of exosome research has developed rapidly. Exosomes contain various cargos, such as proteins, lipids, and nucleic acids, including messenger RNAs, noncoding RNAs, and DNA [4]. By exchanging functional contents between cells, exosomes play fundamental roles in maintaining homeostasis and combatting stress. Many studies have shown that exosomes are involved in a variety of tumor-promoting activities, including anti-apoptosis, metastasis, angiogenesis, immune evasion and chemoresistance. These functions have been well reviewed from various perspectives, such as the type of donor cells or category of contents [5–9]. However, the molecular mechanisms of exosome biogenesis, especially how these mechanisms are exploited by cancer cells, has been less intensively investigated.

**Fig. 1:**
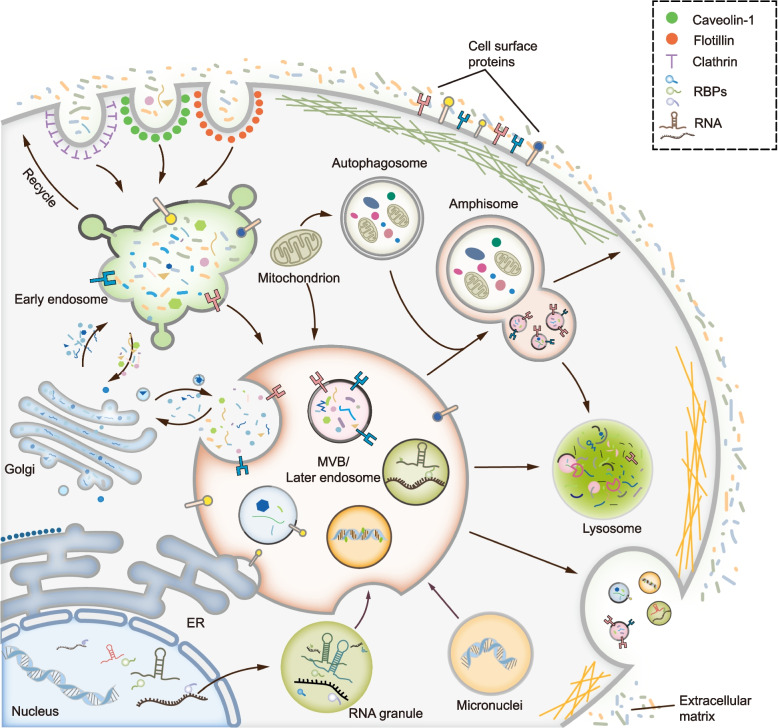
Overview of the process for exosome biogenesis. MVBs take the center of exosome biogenesis. Generally, MVBs are derived from endocytosis, during which different mechanisms mediate the inward budding of the plasma membrane and the formation of early endosomes. MVBs can dynamically communicate with other organelles or compartments including trans-Golgi network (TGN), endoplasmic reticulum (ER), mitochondrion, phagosome, RNA granule and micronuclei, et al. Therefore, different repertoires of cargos such as proteins, RNAs, DNAs or lipids are sorted into MVBs. After the maturation of MVBs, they can either fuse with lysosome to be degraded or fuse with plasma membrane to release ILVs, the so-called exosomes. Of note, MVB can fuse with autophagosome to form amphisome, which can either fuse with lysosome to be degraded or fuse with plasma membrane to secret exosomes

Generally, MVBs are derived by endocytosis, during which multiple mechanisms mediate the inward budding of the plasma membrane and the formation of early endosomes. After recycling a subset of proteins back to the plasma membrane, early endosomes subsequently incorporate various cargo into intraluminal vesicles (ILVs) to generate MVBs. Following their maturation, MVBs can dynamically communicate with other organelles by various paths, such as releasing and taking up vesicles from the trans-Golgi network (TGN) and making direct contact with the endoplasmic reticulum (ER), mitochondrion, or phagosome [10–13]. All these communications modulate the formation of MVBs and the molecular composition of ILVs. Eventually, mature MVBs either fuse with lysosomes to be degraded or fuse with the plasma membrane to release ILVs, so-called exosomes (Fig. 1).

Briefly, exosome biogenesis consists of four steps: cargo sorting to MVBs, MVB formation, transport of MVBs, and MVB-plasma membrane fusion. Each step of exosome biogenesis is mediated by multiple mechanisms, which are highly variable depending on cargo, cell type, and microenvironment and which lead to the heterogeneity of exosomes. In particular, distinct mechanisms are not mutually exclusive, but can be employed by the same MVB. The same type of cargo can adopt different mechanism to mediate exosome sorting. Importantly, cancer cells can exploit multiple strategies to modulate exosome biogenesis and change the composition and function of exosomes, thereby favoring the release of tumor-promoting exosomes.

Herein, we summarize recent progress in understanding the machinery of exosome biogenesis and how this machinery is regulated in cancer. In addition, we provide a comprehensive overview of strategies and inhibitors targeting exosome biogenesis and highlight the perspective of pharmacological targeting of exosome generation in cancer therapy. Throughout, we highlight the key, perplexing questions that prevent us from understanding exosome biogenesis and limit the therapeutic implications of targeting exosome biogenesis.

### The machinery of MVB formation

**Fig. 2:**
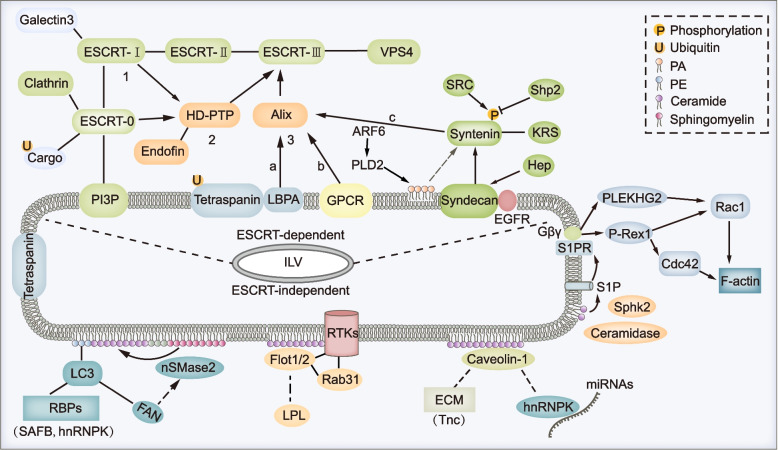
Multiple mechanisms regulate the formation of ILVs. MVBs are characterized by containing intralumenal vesicles which can be controlled by multiple mechanisms. Generally, they can be divided into two categories which are ESCRT-dependent pathway and ESCRT-independent pathway. For the classical ESCRT-dependent pathways, ESCRT-0, -I, -II, -III subcomplexes and ATPase VPS4 cooperate in a stepwise manner to mediate the ILV formation (1). For the non-canonical ESCRT-dependent pathways, HD-PTP (2) or Alix (3) can both recognize specific cargos and recruit ESCRT-III and VPS4 to limiting membrane of MVBs, during which other ESCRT subcomplexes are not indispensable. In addition, several other proteins or pathways especially components of lipid rafts paly crucial roles in ESCRT-independent ILV formation. For example, CD63 could promote ILV formation by both ESCRT and ceramide-independent mechanism; nSMase2-ceramide pathway could drive ILV formation and MVB sorting of cargos such as PE-conjugated LC3 and its binding partners in an ESCRT-independent mechanism. Moreover, caveolin-1 or flotillins could drive lipid raft dependent ILV formation, during which process nSMase-ceramide pathway is required in some cell lines. Specially, F-actin formation on the limiting membrane of MVBs that was regulated by S1P signaling promotes ILV sorting of cargos, though the precise role of F-actin formation during the generation of ILVs is still elusive

MVB formation is at the center of exosome biogenesis. Particularly, membrane budding and ILV generation are key activities in this process [14]. Until recently, multiple mechanisms were proposed to drive the budding of limiting membrane and generation of ILVs (Fig. 2). Generally, the mechanisms consist of endosomal sorting complex required for transport (ESCRT)-dependent and ESCRT-independent pathways.

### ESCRT-dependent pathway

ESCRT is well studied and best described for its function in membrane budding and ILV formation [15]. ESCRT consists of ESCRT-0, -I, -II, -III subcomplexes and the ATPase VPS4, which cooperate in a stepwise manner. ESCRT-0 composed of Hrs and STAM recognizes mono- or poly-ubiquitylated cargo proteins via their ubiquitin-binding domain. In addition, the FYVE domain of ESCRT-0 binds endosomal lipid phosphatidylinositol 3-phosphate (PI3P) through which to capture cargos to clathrin-coated microdomain on the limiting membrane. Subsequently, ESCRT-I and -II are recruited, and they cooperate to form a saddle-shaped protein complex that is important for the ESCRT-III assembly [16]. Following ATP hydrolysis by VPS4, ESCRT-III subunits undergo sequential polymerization and drive membrane deformation and fission to ultimately produce ILV [17]. Notably, the ESCRT complex recruits deubiquitinating enzymes that remove ubiquitin from cargo proteins accompanied by their incorporation into ILVs. But deubiquitylation is not required for all cargos [18].

Several alternative mechanisms, so-called non-canonical ESCRT-dependent pathways, recruit ESCRT-III and ATPase VPS4 to capture cargos into ILVs. Alix and HD-PTP are two auxiliary components of the ESCRT machinery that mediate the alternative mechanisms.

### Alix-dependent pathway

Alix can recruit and nucleate ESCRT-III onto the limiting membrane of MVBs, and recognize cargos in three different ways. The most well-studied Alix-dependent mechanism is the Syndecan-Syntenin-Alix pathway. Syndecan is a ubiquitous transmembrane protein that interacts with adaptor protein Syntenin. Both Syndecan and Syntenin recognize cargos such as fibroblast growth factor receptor (FGFR) and lysyl-tRNA synthetase (KRS) in a ubiquitin-independent manner [19, 20]. Majer et al. reported that membrane protein UNC93B1 recruited Syntenin to facilitate ILV/exosome sorting of UNC93B1-TLR7 complex [21]. After binding to Syntenin, Alix recruits ESCRT-III and VPS4 to complete ILV’s formation [20]. Notably, heparanase can accelerate Syndecan-Syntenin-Alix pathway by trimming the heparan sulfate chains of Syndecan [22]. Besides, the small GTPase ADP ribosylation factor 6 (ARF6) and its effector phospholipase D2 (PLD2), Ral GTPase, SRC tyrosine kinase and phosphatase Shp2 also modulate Syndecan-Syntenin-Alix pathway mediated ILV formation by regulating either the production of phosphatidic acid (PA) or phosphorylation of Syntenin [23, 24]. As the simplest phospholipid, PA directly binds to Syntenin and is speculated to drive negative membrane budding [25]. In addition, Alix directly interacts with lysobisphosphatidic acid (LBPA, also known as BMP) which specifically locates on and contributes to the formation of late endosomes. Concurrently, Alix binds to tetraspanins including CD9, CD63 and CD81 and promotes the sorting of these proteins into ILVs/exosomes [26]. Lastly, Alix binds to the YPX(3)L motif of protease-activated receptor 1 (PAR1) and purinergic receptor P2Y_1_, two G protein-coupled receptors (GPCRs), and mediates their ILV sorting and lysosome degradation in a ubiquitin-independent manner [27].

### HD-PTP-dependent pathway

Several ubiquitinated cargos such as epidermal growth factor receptor (EGFR) and integrins can be recognized by ESCRT-0. After binding to ESCRT-0 or -I, HD-PTP recruits ESCRT-III and VPS4 to complete ILV formation. Eventually, these ubiquitinated cargos are sorted into MVBs and degraded by lysosome, during which interaction between HD-PTP and Endofin is proposed to be required [28, 29].

### Other pathways

Other components of ESCRT can bind and sort cargos into ILVs. For example, by way of the ubiquitin E2 variant domain, Tsg101 (a member of ESCRT-I) specifically recognizes P(S/T)AP sequences in galectin-3 and BAG6, and mediates their ILV/exosome sorting [30, 31]. Of note, members of ESCRT-III can interact with cargos (such as β-catenin) and regulate their transport and exosome release, yet whether there is a direct interaction between them needs to be resolved [32].

### ESCRT-independent pathway

Exosomes are rich in cholesterol, sphingolipids, phosphatidylserine, and ceramide, a composition which resembles that of membrane lipid rafts (or microdomains). In addition, several exosomal proteins, such as flotillins and caveolins, are important components of lipid rafts [33, 34]. Lipid rafts have various functions in protein sorting, membrane curvature, and vesicle budding. Accumulating evidence indicates that components of lipid rafts have key functions in ESCRT-independent ILV formation.

### The nSMase2-ceramide-dependent pathway

The neutral sphingomyelinase 2 (nSMase2)**-**ceramide pathway is the most studied ESCRT-independent mechanism. The nSMase2 is a key enzyme to convert sphingomyelin to ceramide By blocking nSMase2 using the inhibitor GW4869, knocking down nSMase2 expression or treatment with C6-ceramide, the nSMase2-ceramide pathway has been demonstrated to control ILV/exosome sorting of multiple cargos, such as proteolipid protein (PLP) in oligodendroglia cells, the prion protein in neuronal cells, and several RNAs in cancer cells [35–37]. FAN, a WD-repeat protein, was found to enhance nSMase2 activity and promote ceramide production [38]. In HEK293T and several cancer cells, phosphatidylethanolamine-conjugated LC3 located on MVBs interacts directly with and recruits FAN to the limiting membrane. Subsequently, cargos containing LC3-interaction region, such as RNA-binding proteins SAFB and hnRNPK, are incorporated into MVBs through nSMase2-ceramide-dependent pathway, which process is distinct from classical autophagy and is independent of the ESCRT-mediated ILV formation [39] (Fig. 2). Nonetheless, unlike the ESCRT-dependent pathway, the exact mechanism of ILV formation mediated by the nSMase2-ceramide-dependent pathway has not been clearly elucidated. Ceramide can induce the formation of large macrodomains on the plasma membrane by its hydrogen bonding-dependent self-association. Then, the macrodomain functions as a platform to trap other molecules such as CD95 and CD40 [40, 41]. Also, ceramide can induce budding and formation of small vesicles in vitro. Therefore, it was proposed that a cone-shaped structure formed by ceramide induced spontaneous negative curvature of the membrane [35]. Clarification is needed as to whether ceramide cooperates with other molecules to promote the deformation and scission of limiting membrane of MVBs in vivo. In particular, other components of lipid rafts are proposed to be involved in ILV formation, described as follows.

### Caveolin-1

Caveolin-1 is a hairpin-like integral membrane protein that binds cholesterol on membrane. Acting as a scaffold to assemble lipids and proteins, caveolin-1 initiates formation of caveola on the plasma membrane and mediates caveola-dependent endocytosis [42] (Fig. 1). When caveolae assembly is compromised, caveolin-1 is internalized and sorted into ILVs, then degraded in lysosome. This degradation is ubiquitin- and ESCRT machinery-dependent [43]. Conversely, caveolin-1 can also be released into exosomes. For example, ubiquitinated caveolin-1 is sorted to MVBs, where it centrally regulates ILV/exosome sorting of extracellular matrix cargos such as Tenascin-C. The nSMase2-ceramide pathway is a major limiting step in this caveolin-1-mediated cargo sorting and exosome biogenesis, because knocking down TSG101 also has a weak effect on the release of exosomal Tenascin-C [44]. In addition, caveolin-1 regulates translocation of hnRNPK to lipid rafts on the limiting membrane of MVBs, whereby hnRNPK together with its bound microRNAs are sorted into ILVs/exosomes in PC3 prostate cancer cells [45] (Fig. 2). However, caveolin-1 does not interact directly with hnRNPK. Thus, further study is needed to investigate how hnRNPK is captured by lipid raft on limiting membrane and whether the LC3-conjugation system is involved in this process. Somewhat controversially, although the nSMase2-ceramide pathway is required for caveolin-1-mediated exosomes generation in mouse embryonic fibroblasts and MDA-MB468 breast tumor cells [44], inhibition or depletion of nSMase2 does not affect exosomal secretion of caveolin-1 in PC-3 cells [46]. Thus, caveolin-1 could mediate ILV/exosome sorting of cargos by the ESCRT-independent pathway, and whether the nSMase2-ceramide pathway is involved in this process depends on the cell type.

### Flotillins

Flotillins are membrane scaffolding proteins that participate in the formation of lipid rafts and are involved in a wide range of cellular events such as endosome trafficking and protein sorting [47]. Flotillins, especially flotillin-1, have been used as markers of exosomes for nearly 20 years [48]. Particularly, several studies have shown that flotillins control exosome sorting of cargos such as LGI3 and caveolin-1 in specific cell types [46, 49]. Wei et al. proposed that activation of EGFR upon EGF stimulation could activate Rab31 which, in turn, binds directly to flotillins and drives lipid raft-dependent exosome sorting of receptor tyrosine kinases such as EGFR. Both ceramide and cholesterol, but not ESCRT, are required for this process [50] (Fig. 2). Of note, on the plasma membrane, flotillin positive microdomains are distinct from caveolin-1 positive caveolae [51]. Nevertheless, in PC-3 cells, knocking down flotillin-1 reduced exosomal release of caveolin-1 [46]. Thus, whether coveolin-1 and flotillins cooperate to mediate cargo sorting and ILV formation, or whether they might be associated with distinct microdomains on MVBs remains to be determined.

### Cholesterol

Cholesterol is enriched in ILVs/exosomes and is involved in the localization and trafficking of late endosomes. Cholesterol can be synthesized de novo in the endoplasmic reticulum (ER) or it can be ingested exogenously via endocytosis [52]. At the intersection of these two routes, ER-endosome contact sites have an important function in modulating the cholesterol content on MVBs [53]. Specifically, endosomal annexin A1 interacts with ER-localized S100A11 to mediate contact site formation, which promotes interaction between PTP1B and cargos on MVBs, such as Hrs (a component of ESCRT-0) and EGFR. This process accelerates cholesterol transfer from ER to MVB, which is mediated by the lipid transfer protein ORP1L. Subsequently, ESCRT and cholesterol cooperatively contribute to formation of MVB and sequestering cargos such as EGFR into ILVs [10, 54]. Another lipid transfer protein, STARD3, is involved in ER-endosome contact site formation and cholesterol transport from ER to MVBs, which promotes ILV formations in HeLa cells [53]. Except for its function in ILV formation, cholesterol also has an effect on exosome biogenesis. Strauss et al. and Zhao et al. proposed that cholesterol promoted exosomal secretion of flotillin 2 and miR-122-5p in Oli-neu oligodendroglial cells and Huh7 hepatocellular carcinoma cells, respectively [55, 56]. Controversially, knocking down ORP1L promotes Tenascin-C incorporation into MVBs accompanied by reduced cholesterol content in fibroblasts. Also, treatment with U18666A, which increases cholesterol in MVBs, reduces ILV sorting and exosome release of Tenascin-C, yet total exosome secretion is enhanced in fibroblasts [44]. Additionally, cholesterol negatively regulates the exosomes release in astrocyte in vitro and in vivo [57]. Thus, cholesterol seems to exert opposite effects in the formation of ILVs/exosomes, which might vary depending on cell type or cargo.

### Tetraspanins

Tetraspanins are a family of membrane scaffolds that incorporate proteins into Tetraspanin-enriched microdomains, thereby controlling signal transduction and a variety of cellular activities [58]. Increasing evidence has shown that Tetraspanins contribute to ILV sorting of various cargos. For instance, CD63 coupled with Apolipoprotein E promotes ILV formation and mediates ILV sorting of melanocyte protein PMEL via both ESCRT and ceramide-independent manner. Consistently, knocking down or knocking out CD63 reduces ILV formation and exosome biogenesis [59]. Several other cargos such as oncogene latent membrane protein 1 (LMP1), vascular endothelial growth factor (VEGF), ferritin and some nuclear content are also proposed to be sorted into ILVs/exosomes via CD63-dependent mechanism [60–62]. Additionally, Tetraspanin-6 (TSPN6) interacts with Syntenin to promote ILV sorting and exosomal secretion of amyloid precursor protein in HEK293 cells [63]. Paradoxically, TSPN6 could directly mediate lysosomal degradation of Syndecan-4 and inhibit exosome generation in MCF-7 cells [64]. Therefore, TSPN6 might have distinct functions in different cell models or for different cargo. Of note, for most of Tetraspanins-mediated ILV formation discussed above, the role of ESCRT and ceramide was not investigated.

### Others

There are several other ESCRT-independent mechanisms that are proposed to mediate cargo sorting and ILV formation. For instance, under the sequential action of ceramidase and sphingosine kinase 2 (SphK2), ceramide can be catabolized to sphingosine-1-phosphate (S1P) which binds to and activates its receptor S1PR. Subsequently, S1PR promotes the interaction between β and γ subunits of G protein with P-Rex1 and PLEKHG2 which are GEFs for Rac and Cdc42. Eventually, activation of Rac and Cdc42 contributes to formation of F-actin, thereby promoting ILV sorting and exosome release of cargos such as CD63, CD81 and flotillins [65, 66] (Fig. 2). Ekström et al. found that Wnt5a could activate its effector Cdc42 and promote the biogenesis of exosomes containing pro-angiogenic and immunosuppressive cargos [67]. However, compared with the mechanism of F-actin formation, the exact function of F-actin in the formation of ILVs is unclear.

Collectively, multiple mechanisms mediate the formation of ILVs/exosomes and most of these mechanisms are not completely understood. For example, PD-L1 binds with Hrs and can be sorted into exosomes in MEL624 melanoma cells [68]; however, knocking down either Hrs or nSMase2 blocks exosomal secretion of PD-L1 [69]. In addition, down-regulating ceramide reduces the exosomal level of TSG101 in GT1–7 mouse hypothalamic neuronal cell line and inhibits Syndecan-Syntenin-Alix mediated generation of flotillin-1-negative exosomes in MCF-7 cells [20, 36]. In particular, ESCRT-dependent or -independent pathways can function on the same MVB and therefore distinct populations of ILVs enriched with different cargos are produced within a single MVB [35, 59, 70, 71]. Thus, ESCRT-dependent and -independent pathways are not mutually exclusive but intersect to some extent, which might depend on cell type, cargo, and cellular contexts.

### Cargo sorting to MVBs

Sorting to MVBs is indispensable for the secretion of exosomal cargo. In addition to lipids, exosomes contain a variety of proteins and nucleic acids including messenger RNAs (mRNAs), microRNAs (miRNAs), long non-coding RNAs (lncRNAs), circular RNAs (circRNAs), PIWI-interacting RNAs (piRNAs) and DNA [4]. Depending on the state of source cells, exosomal content profiles change dynamically and determine functional properties of exosomes [72, 73]. Therefore, packaging of cargos into exosomes is highly selective and is under tightly regulated.

### Proteins

Proteins can be sorted into exosomes by a variety of pathways. Many proteins are sorted into MVBs by directly interacting with ESCRT-dependent or ESCRT-independent machinery as described earlier. Some cargos, especially cytoplasmic proteins, can also be selectively sorted into ILVs/exosomes. For example, Hsp90α can be sorted into exosomes by its interaction with Rab coupling protein [74]. Fusion protein Rab22a-NeoF1 and its binding partner PYK2 can be sorted into MVBs/exosomes via interaction with Hsp90 in ESCRT-dependent pathway [75]. Cytosolic Ago2 can be co-sorted into ILVs/exosomes by its association with Alix [76]. In addition, chaperone Hsc70 sorts cytosolic proteins to ILVs/exosomes [77]. Recently, Ferreira et al. showed that the KFERQ motif-containing proteins such as HIF1α are loaded into ILVs/exosomes by directly binding to LAMP2A and Hsc70, which process depends on Alix, Syntenin-1, Rab31 and ceramides rather than the ESCRT machinery [78].

### RNAs

Generally, RNA-binding proteins (RBPs) that contain sequence-specific RNA-binding domain have a key function in exosomal sorting of RNAs [79]. Many RBPs, such as hnRNPA2B1, hnRNPK, YBX1, major vault protein (MVP), MEX3C, synaptotagmin-binding cytoplasmic RNA-interaction protein (SYNCRIP), Ago2 and FMR1, are proposed to participate in the ILV/exosome sorting of miRNAs in different cell models [45, 80–83]. As for lncRNAs, hnRNPA2B1 and hnRNPA1 play a role in exosome loading of lncARSR, LNMAT2 and ELNAT1 [84, 85]. Pan et al. and Chen et al. proposed that SNF8, a subunit of ESCRT-II, and hnRNPA2B1 mediated exosomal sorting of circRNAs including circRHOBTB3 and circNEIL3 in glioma and colorectal cancer, respectively [86, 87]. The exact mechanism through which RBPs are recruited to the limiting membrane of MVBs and subsequently incorporated into ILVs is largely unknown. As described earlier, caveolin-1 and the LC3-conjugation machinery mediate the MVB sorting and exosomal release of RBPs including hnRNPK [39, 45]. However, many RBPs such as hnRNPA2B1 and hnRNPK are located mainly in the nucleus; how they are translocated into cytoplasm and sorted into MVBs is yet to be determined.

Liquid–liquid phase separation (LLPS) is a ubiquitous physical process by which membraneless compartments/condensates such as RNA granules (e.g., stress granules and P-bodies) are formed in cells [88]. RBPs and RNAs have pivotal functions in LLPS-mediated condensate formation [88, 89]. For the exosomal secretion mediated by the LC3-conjugation machinery, many target cargos are components of RNA granules such as hnRNPK and SAFB. Both of these cargos and LC3 colocalize with Rab5-marked MVBs [39]. In addition, hnRNPA2B1, which contributes to exosomal sorting of miRNAs, lncRNAs and circRNAs, also undergoes LLPS and is a component of RNA granules [90]. Therefore, it is attractive to ask whether and how LLPS contributes to exosomal release of RBPs and RNAs and whether RNA granules act as “relay station” during this process. In studies of the RNA-binding protein YBX1, the Schekman team provided some answers to these questions. YBX1 was proposed to interact with and mediate exosomal sorting of miRNA-223 via ceramide-dependent pathway. Recently, the Schekman team reported that YBX1 undergone LLPS to form condensates, thereby selectively recruiting and sorting miR-223 into exosomes, a process that was inhibited by perturbing phase separation of YBX1 [91]. Although the mechanism whereby YBX1 condensates are captured and invaginated into MVBs was not established, LLPS indeed functions directly in the exosomal sorting of RBPs and their associated microRNAs. Notably, structured RNAs such as lncRNAs play key roles in LLPS [88, 92]. Further study is needed to determine whether these RNAs cooperate with RBPs to form condensates and actively contributes to their exosomal sorting.

### DNA

Contradictory conclusions exist for exosomal sorting of DNA molecules. Jeppesen et al. showed that extracellular secretion of DNA and histones was mediated by exosome-independent mechanisms [93]. Conversely, Takahashi et al. and Torralba et al. found that gDNA and some nuclear proteins were sorted into exosomes [94, 95]. In cancer cells such as ovarian cancer, micronuclei containing gDNA and nuclear proteins interact with Tetraspanins such as CD63 by which they are sorted into ILVs/exosomes [61] (Fig. 1). In addition, mitochondrial DNA was reported to be released into exosomes [12]. Mechanistically, PINK1 activated by mitochondrial damage mediates interaction between MVBs and mitochondria. And, mGluR3 promotes Rab27-dependent antegrade transport of these MVBs and section of exosomes which drive invasiveness in breast cancer cells. Of note, the sorting of mitochondrial cargos into MVBs is LC3/autophagy-independent; how the mitochondrial chromosome is transferred into ILVs and whether ESCRT or other mechanisms contribute to this process is yet to be determined.

### Maturation and fate of MVBs

MVBs (also referred to as multivesicular endosomes, MVEs) are defined by their morphology observed by electron microscope and are characterized as containing intralumenal vesicles [96]. Maturation of MVBs is a complicated process during which MVBs fuse with each other or communicate with other organelles such as the ER, TGN, mitochondria, recycling endosome, and RNA granules [11, 12, 91, 97, 98] (Fig. 1). Under particular conditions, autophagosomes fuse with MVBs to form amphisomes which are special kind of mature MVB. Similarly, amphisomes can either fuse with lysosome to be degraded or fuse with plasma membrane to secret exosomes. The mechanism for the formation of amphisomes is excellently reviewed elsewhere [13]. In addition, another special MVB known as recycling endosomal MVB and marked by Rab11 (an acknowledged marker of recycling endosome) is proposed to exist in a variety of cancer cells. Recycling endosomal MVBs are distinct from CD63 positive MVBs/late endosomes. And, glutamine depletion or mTORC1 inhibition promotes the generation of the recycling endosomal MVBs and secretion of Rab11-positive exosomes [99]. More recently, Arya et al. found the nuclear envelope derived MVBs in activated neutrophils. These MVBs are 5-lipoxygenase (5-LO)/5-LO activating protein (FLAP) positive and are distinct from CD63 positive MVBs in both size and composition [100]. Therefore, MVBs are highly heterogeneous according to their origination, maturation state or route. Considering the diversity of exosomal content and mechanisms of ILVs formation, conceivably, additional subpopulations of MVBs might be present in a single cell to generate heterogeneous population of exosomes.

**Fig. 3:**
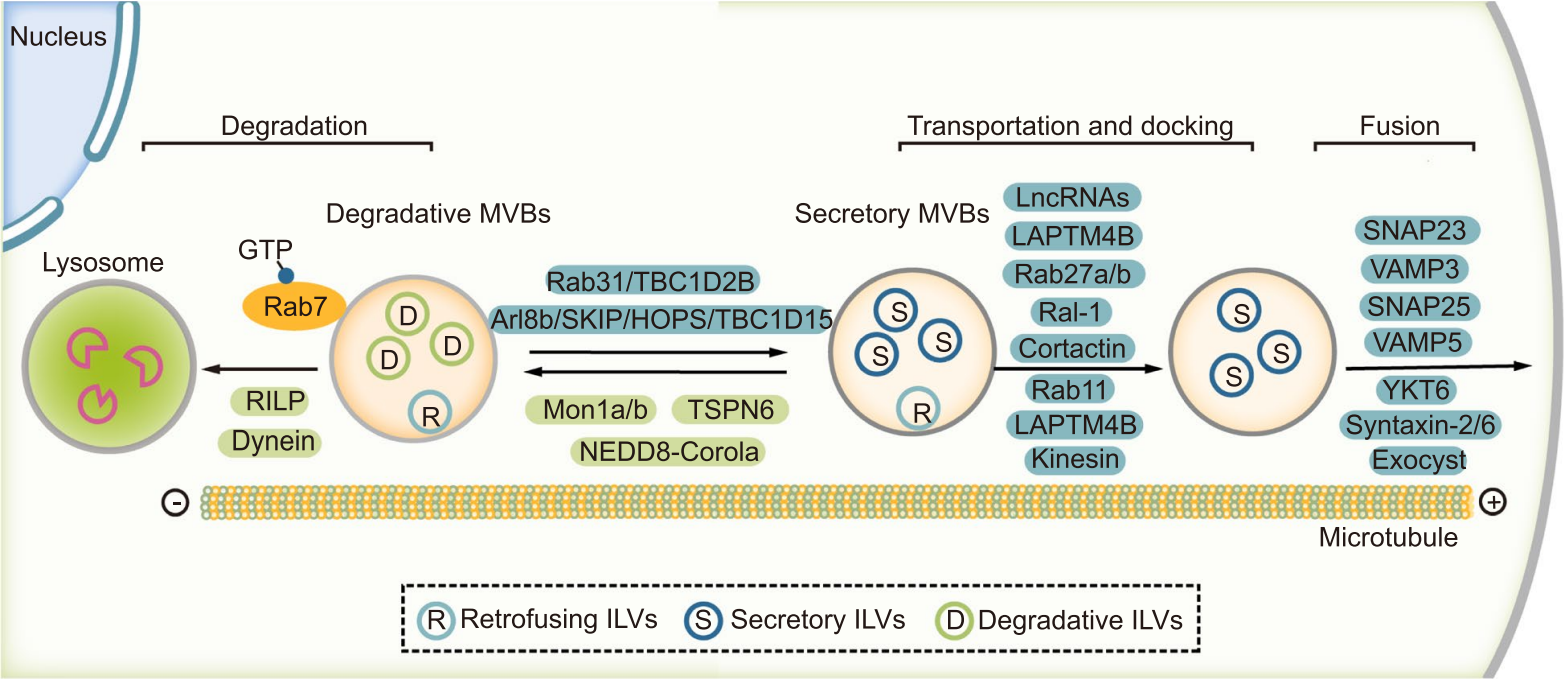
Mechanisms mediating the transport and fate of MVBs. After the maturation of MVBs, they can either fuse with lysosome or fuse with plasma membrane. The activity of Rab7 plays a pivotal role for the fate of MVBs. Mon1a/b and neddylated Coro1a can activate Rab7 and promote dynein-dependent retrograde transport of MVBs towards the minus-end or perinuclear region. On the other hand, Arl8b/SKIP/HOPS/ TBC1D15 cascade or Rab31/TBC1D2B cascade could inactivate RAB7 and promote kinesin dependent antegrade transport of MVBs towards the plus-end or cell periphery. The antegrade transport of MVBs and their docking, tethering and fusion with plasma membrane are controlled by multiple factors including both proteins and lncRNAs. Notably, after the formation of MVBs, the ILVs inside can still retrofuse with the limiting membrane of MVBs and these ILVs are referred as retrofusing ILVs (rILVs). The others are recognized as secretory ILVs (sILVs) or degradative ILVs (dILVs)

According to their fate, MVBs degraded by fusion with lysosomes are referred as degradative MVBs (dMVBs); MVBs fusing with the plasma membrane are secretory MVBs (sMVBs). The activity of Rab7 is proposed to be pivotal for the fate of MVBs/late endosomes (Fig. 3). Guanine nucleotide exchange factor (GEF) and GTPase activating proteins (GAPs) control the active GTP bound state and inactive GDP bound state of the Rab GTPase, respectively. Jongsma et al. proposed that the Arl8b/SKIP/HOPS cascade recruits a GAP TBC1D15to inactivate and remove Rab7 from the MVBs/late endosomes. By the action of kinesin motors, the MVBs/late endosomes subsequently move toward the plus-end or cell periphery. Alternatively, RILP, the effector of Rab7, mediates the dynein-dependent retrograde transport of MVBs/late endosomes towards the minus-end or perinuclear region in HeLa cells [101]. Wei et al. showed that Rab31 recruits the GAP TBC1D2B to inactivate RAB7, thereby inhibiting the degradation of MVBs but promoting their fusion with plasma membrane and exosome secretion in HeLa and HEK293T cells [50]. Conversely, Mon1a/b and neddylated Coro1a act as GEFs to activate Rab7 and inhibit exosome biogenesis in HeLa and HEK293T cells [102]. In addition, inhibiting lysosomal function or autophagy promotes exosome secretion [103]. Thus, the fate of MVBs are regulated by multiple mechanisms and inactive Rab7 contributes to the formation of sMVBs. However, knocking-down Rab7 in MDA-MB-231 breast cancer cells severely reduced the Syntenin-Syndecan-Alix dependent exosome secretion in MCF7 cells [20]. Knocking down Rab7 in HCT116 colorectal cancer cells inhibits total exosomes release but promotes the secretion of Rab11a positive exosomes [99]. Moreover, Rab7 is indispensable for the ER-endosome contact mediated secretion of late endosomes/lysosomes [104]. Therefore, Rab7 might play multi-roles in the formation and fate of MVBs/late endosomes.

Notably, ILVs can return and retro-fuse with the limiting membrane of MVBs in parental cells, a mechanism by which some proteins can be recycled back to the limiting membrane. These ILVs that can retro-fuse with the limiting membrane of MVBs are recognized as retrofusing ILVs (rILVs). Interestingly, ILVs destined for secretion as exosomes are inert to retro-fusion and these ILVs are referred as secretory ILVs (sILVs). Namely, sMVBs contain both sILVs and rILVs, and dMVBs contains degradative ILVs (dILVs) and rILVs [105] (Fig. 3). However, the exact mechanism that controls retrofusion or the fate of ILVs is unknown.

### Transport of MVBs

After the maturation of sMVBs, several mechanisms act sequentially or concomitantly to control sMVBs antegrade movement, tethering/docking, and fusion with the plasma membrane (Fig. 3).

### Rab GTPases

**Fig. 4:**
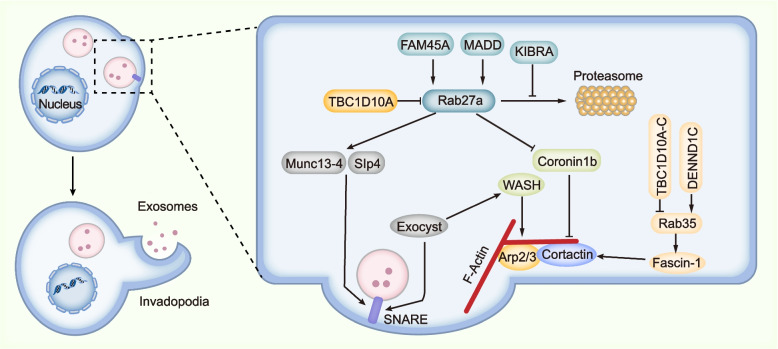
Proposed model showing the relationship between MVB secretion, actin reorganization and invadopodia formation. These three processes are highly organized and interrelated. Specifically, invadopodia determines the docking and secretion sites of MVBs on the plasma membrane. On the other hand, the fusion of MVBs on the plasma membrane contributes to the formation of invadopodia. And, F-actin formation is vital for both MVB secretion and invadopodia formation. Mechanistically, Rab27a and Rab35 seem to function at the center, the activity of which is regulated by their GAPs or GEFs. Particularly, Rab27a promotes both MVB docking and F-actin formation. Munc13-4 and Slp4 are effectors of Rab27a and function to mediate the docking and fusion of MVBs on the plasma membrane by promoting the formation of SNARE. Concurrently, Rab27a inhibits Coronin1b binding to invadopodia-associated actin and stabilizes Cortactin-mediated branched actin. In addition, actin-binding protein Fascin-1 is an effector of Rab35 and contributes to both invadopodia and exosome secretion. Moreover, exocyst complex binds to WASH, through which to promote Arp2/3 mediated actin polymerization and invadopodia formation. Also, exocyst binds to SNARE and mediates docking and fusion of MVBs on the plasma membrane

Rabs, a family of small GTPases that contains approximately 70 members in human, play a key role in the transport and fate of intracellular vesicles [106]. Based on RNA interference (RNAi) screen in Hela cells, many Rabs are proposed to regulate exosome biogenesis, including Rab2b, Rab9a, Rab5a, Rab27a and Rab27b but not Rab7, Rab11a and other Rabs [107]. Mounting evidence has shown that Rab27a/b along with their upstream regulators and downstream effectors play a fundamental role in exosome biogenesis (Fig. 4). Specifically, Rab27a and its effector Slp4 function in docking of MVBs on plasma membrane, and Rab27b and its effector Slac2b mediate the transfer of MVBs from the perinuclear area to the cell periphery [107]. Slp4 (also known as granuphilin) interacts with Rabs via its N-terminal Slp homology domain and binds to SNARE complex via a central linker region [108]. Park et al. reported that disrupting the interaction between Rab27a and Slp4 with inhibitor BHMPS reduced exosome secretion and inhibited tumor growth of breast cancer cells [109]. Munc13-4 is another effector of Rab27. As a Ca^2+^-dependent SNARE- and phospholipid-binding protein, Munc13-4 interacts with several Syntaxins including Syntaxin-1/2/4/7/11 which are components of SNARE complexes, thereby regulating membrane fusion and exocytosis [104, 110–112]. Additionally, DENN domain-containing protein Rab3GEP (also known as MADD) and FAM45A are GEFs of Rab27 and contribute to exocytosis in some cell types [113, 114]. TBC1D10A (or EPI64) is a GAP of Rab27a and controls the transport of melanosomes in mouse melanocytes [115]. Moreover, KIBRA interacts with Rab27a, inhibits its proteasomal degradation and subsequently promotes exosome secretion in neuronal and podocyte cell [116]. Notably, Rab27b inhibits exosomal sorting of PD-L1 in hepatocellular carcinoma by diverting PD-L1 from endosomes in the TGN area to the plasma membrane and this effect of Rab27b can be suppressed by GOLM1 [117]. Thus, Rab27b has different functions in regulating exosome secretion in different cells or for different cargos.

By performing the proteome screen of purified exosomes from murine oligodendroglia, Hsu et al. proposed that Rab35 mediated the docking or tethering of MVBs with the plasma membrane to promote exosome secretion, which was inhibited by TBC1D10A-C, GAPs of Rab35 [118]. Besides, Rab11 and its effector Munc13-4 mediates the fusion of recycling endosome with MVBs in MDA-MB-231 cells, thereby promoting MVB translocation and exosome secretion of membrane type 1 matrix metalloproteinase (MT1-MMP), which is essential for cancer metastasis [98]. Savina et al. proposed that Rab11 mediated homotypic fusion of MVBs and promoted docking and fusion of MVBs to the plasma membrane in K562 cells [97]. In addition, Rab11a regulates exosome secretion and cell proliferation of HN4 head and neck carcinoma cells by interacting with the exocyst complex which interacts with SNARE and mediates the MVBs-plasma membrane fusion event [119]. Except for Rab31 as described earlier, other Rabs such as Rab17 and Rab20 are also proposed to be involved in exosome generation [120, 121]. Recently, Matsui et al. showed that Rab27a and its homolog Rab37 specifically mediated the exosome release on apical membrane of polarized Madin-Darby canine kidney (MDCK) epithelial cells. On the other hand, Rab39 along with its effector UACA recruited the BLOC-1 related complex (BORC) to mediate antegrade MVB transport and exosome secretion on basolateral membrane [122]. Collectively, Rabs are vital for exosome biogenesis and different Rabs are responsible for the secretion of different exosomal cargos or MVBs in specific cell type.

In addition to Rabs, several other factors are proposed to be involved in the transport of MVBs. For example, Lysosome-associated protein transmembrane 4B (LAPTM4B) and Hsp90 facilitates MVB transport toward the plasma membrane and enhance exosome secretion [123, 124]. In addition, ER-Endosome contact sites mediate transport of Rab7- and LAMP1-positive late endosomes to the plasma membrane and promote invadopodia maturation and MT1-MMP exocytosis [125]. Therefore, multiple mechanisms might act in parallel to regulate the transport of distinct subpopulations of MVBs and their fusion with the plasma membrane.

### Invadopodia formation, actin reorganization and MVB secretion

Invadopodia are protrusion on tumor cells that contribute to tumor cell invasion and dissemination [126]. Notably, invadopodia are specific and critical docking and secretion sites for MVBs, and disrupting invadopodia formation inhibits exosome secretion of proteinases such as MT1-MMP. In turn, the secretion of MVBs also functions in the formation of invadopodia [127, 128] (Fig. 4). Actin reorganization is highly coordinated with the formation of invadopodia, and the Arp2/3 complex plays a key role in nucleation of actin polymerization. By linking Arp2/3 to actin filaments, cortactin mediates MVB docking and exosome secretion in invadopodia, which are coordinately controlled by Rab27a and Coronin1b [129]. In addition, Arp2/3 activator Wiskott-Aldrich syndrome protein and SCAR homolog (WASH) promote actin polymerization and invadopodia formation, and mediate docking and fusion of MT1-MMP-positive MVBs on the plasma membrane via interacting with exocyst complex [130]. Rab35 regulates F-actin organization and invadopodia formation via its effector fascin (Fascin-1 in human), which process is controlled by connecdenn 3/DENND1C, a GEF of Rab35 [131]. Consistently, inhibiting Fascin-1 reduces exosome-like vesicle release in MDA-MB-231 breast cancer cells [132]. Therefore, invadopodia formation, actin reorganization and MVB secretion are highly organized. However, whether all MVBs are secreted on invadopodia or are there other factors determined the site of exosome release on membrane are not yet clarified.

### SNARE

The SNARE complex is composed of three or four SNARE proteins that are located on vesicle and target membrane, and is the executor of membrane fusion. Nearly 40 different SNAREs, the composition of which are spatially and temporally regulated, are involved in different intracellular fusion events [133]. So far, multiple SNAREs differing in composition are proposed to mediate MVB-plasma membrane fusion in different cells. For instance, the Fas/Fap-1/caveolin-1 cascade mediates the formation of SNARE composed of SNAP25 and VAMP5 in bone marrow mesenchymal stem cells [134]. In hepatocellular carcinoma, SNAP23 and VAMP3 are required for the fusion event, which can be regulated by lncRNA HOTAIR [135]. SNARE composed of VAMP7, Syntaxin-4, and SNAP23 has an important function in the fusion of MT1-MMP-positive late endosomes to invadopodia [136]. Moreover, Syntaxin-6 and VAMP3 were proposed to regulate MVB-plasma membrane fusion and exosome release in prostate cancer and neurons, respectively [137, 138]. In Caenorhabditis elegans, the small GTPase RAL-1 mediates fusion of MVBs with plasma membrane; this fusion is dependent on SYX5, a homologue of mammalian Syntaxin-5, but does not require exocyst complex [139].

### Regulation of exosome biogenesis in cancer

**Fig. 5:**
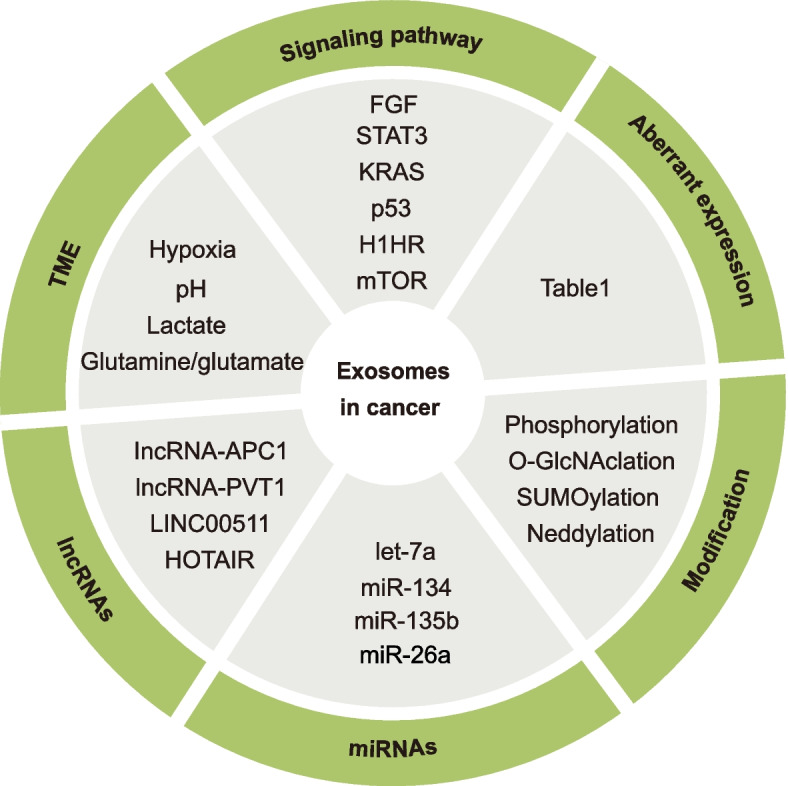
Categories of mechanisms mediating the dysregulation of exosome biogenesis in cancer

Exosomes contribute to cancer cell survival and metastasis by promoting their ability to deal with stresses, develop chemoresistance and evade immune surveillance [5, 6]. Additionally, exosomes are proposed to be involved in the active efflux of waste products or tumor suppressors from cancer cells to maintain cellular homeostasis [94, 140–142]. To achieve these tumor-promoting roles of exosomes, cancer cells exploit various strategies to regulate the machinery of exosome biogenesis (Fig. 5). A better understanding of these strategies will be conducive to unraveling novel targets or approaches for cancer therapy.

### Aberrant expression

Exosomes can be directly modulated in the quantity, composition and functions by aberrant expression of their biogenesis machinery in cancer. Actually, some elements of the machinery are oncogenes or tumor suppressors (Table 1). For the ESCRT-dependent pathway, Hoshino et al. found that oncogene Hrs promoted invadopodia formation and cell invasion in SCC61 HNSCC cells [128]; Zhong et al. reported that Hsp90, ubiquitously activated in cancer, promoted exosomal secretion of Rab22a-NeoF1 fusion protein, thereby promoting cancer metastasis in osteosarcoma cells and several other cancers [74, 75]. Conversely, by mediating exosomal secretion of oncogenic molecules, Vps4A acted as a tumor suppressor and inhibited the proliferation and metastasis of parental cells in hepatocellular carcinoma [32]; HD-PTP exerts its anti-tumor effect by promoting oncogenic protein such as EGFR and integrins into MVBs and accelerating their lysosome degradation but not exosomal secretion in HeLa cells [28, 143]. In the Syndecan-Syntenin-Alix dependent pathway, Syntenin, SRC, heparinase, and Ral act as oncogenes that promote tumor progression and metastasis by their functions in exosome generation [144–148]. Ghossoub et al. proposed that TSPN6 determined the fate of MVBs [64]. Andrijes et al. found that TSPN6 and its adaptor Syntenin-1 negatively mediated the exosomal secretion of the transmembrane form of TGF-α and inhibited carcinogenesis in colorectal cancer [149]. For the ESCRT independent pathway, both caveolin-1, flotillins, and Rab31 are ubiquitously overexpressed in cancer and promote cancer progression and chemoresistance by their function in controlling exosomes secretion [45, 50, 150]. Several RBPs such as hnRNPA2B1, hnRNPK, hnRNPA1, MVP, and hnRNPH1 contribute to tumor progression, metastasis, and immunosuppression by directly or indirectly regulating exosome generation in a variety of cancers [45, 85, 86, 151, 152].

**Table 1:** Genes modulating exosome biogenesis and their roles in cancer

	Genes	Roles	Targets or pathways	Cancer cell types	Refs
Cargo sorting	hnRNPA2B1	Oncogene	miR-122-5p, circNEIL3	NSCLC, glioma	[86, 151]
	hnRNPK	Oncogene	miR-148a-3p	Prostate cancer	[45]
	hnRNPH1	Oncogene	-	Prostate cancer	[152]
	hnRNPA1	Oncogene	lncRNA ELNAT1	Bladder cancer	[85]
	MVP	Oncogene	miR-193a	Colon cancer	[83]
	Hsp90	Oncogene	Rab22a-NeoF1	Osteosarcoma	[75]
ILV formation	Hrs	Oncogene	ESCRT dependent	HNSCC, breast cancer	[128]
	HD-PTP	Suppressor	ESCRT dependent	HeLa cell	[28, 143]
	Vps4A	Suppressor	ESCRT dependent	HCC	[32]
	SRC	Oncogene	Syndecan-Syntenin-Alix	CML, breast cancer	[147]
	Heparanase	Oncogene	Syndecan-Syntenin-Alix	Glioma	[148]
	Ral	Oncogene	Syndecan-Syntenin-Alix	Breast cancer	[146]
	TSPN6	Suppressor	Syntenin dependent	CRC	[149]
	Caveolin-1	Oncogene	ESCRT independent	Prostate and breast cancer	[44, 45]
	Rab31/ Flotillins	Oncogene	ESCRT independent	NCI-H1975, MDA-MB231, and HeLa cells	[50]
	Rab35	Oncogene	ESCRT independent	HCC	[135]
MVB transport and fusion	Rab27a/b	Oncogene	Docking	HNSCC, HCC, cervical, ovarian, breast, Melanoma, bladder and lung cancer	[153, 154]
	Rab17	Oncogene	-	NSCLC	[120]
	Rab7	Oncogene	Syndecan-Syntenin-Alix, ER-endosome contact sites	Breast cancer	[20, 104]
	Cortactin	Oncogene	F-actin reorganization	NSCLC, breast cancer	[129, 132]
	Syt7	Oncogene	MVB-plasma membrane Fusion	NSCLC, breast cancer	[104, 128]
	Munc13–4	Oncogene	MVB maturation	Breast cancer	[98]
	Exocyst	Oncogenes	MVB-PM Fusion	HNSCC	[119]

Note: The name of the cancer cell line is listed here only when no more cell lines of the same cancer type were used

*CML* Chronic myeloid leukemia, *HNSCC* Head and neck squamous cell carcinoma, *HCC* Hepatocellular carcinoma, *NSCLC* Non-small cell lung cancer, *CRC* Colorectal cancer, *OSCC* Oral squamous cell carcinoma

The mechanism of MVB transport and fusion with the plasma membrane can also be exploited by cancer. Rab27a/b are aberrantly expressed in cancers, and they have a key function in cancer progression via their activity in exosome biogenesis [109, 153]. In addition, depletion of elements that mediates ER-endosome contact sites or invadopodia formation, such as Rab7, Protrudin, FYCO1, fascin-1, and cortactin, blocks exosome biogenesis and inhibits invasion in NSCLC and breast cancer [104, 129, 132]. And, Rab7, fascin-1, and cortactin are proposed to be oncogenes [155, 156]. Other factors such as LAPTM4B and PLD2 are overexpressed in a variety of cancers and promote cancer proliferation, invasion and metastasis; yet it is not known whether and how they exert cancer progression activities within the exosomal pathway [157].

### Tumor microenvironment

Hypoxia, decreased extracellular pH, and high concentration of lactate are common characteristics of the tumor microenvironment (TME), which is important for cancer cell survival, metastasis, immune evasion and chemoresistance [158, 159]. These characteristics modulate exosome generation in both cancer cells and some other cell types within the TME, through which to favor tumor progression.

### Hypoxia

Hypoxia modulates the secretion, composition, and function of exosomes in various cancers [160, 161]. Dorayappan et al. found that hypoxia increased exosome release by upregulating Rab27a and reducing Rab7, LAMP1/2, and NEU-1, thereby promoting cell migration/invasion and chemoresistance in vitro and in vivo in ovarian cancer cells [154]. In addition, oxidized ATM that was induced by hypoxia phosphorylated BNIP3 and ATP6V1G1, both of which contribute to autophagy-associated exosome release in cancer-associated fibroblasts (CAFs). Xi et al. showed that exosomes secreted by these CAFs promoted migration and invasion of breast cancer cells [162]. Notably, hypoxia inducible factor 1 (HIF1) was proposed to contribute to exosome generation, but the underlying mechanism remains unclear [163].

### Low pH

Low pH promotes secretion of exosomes that contain caveolin-1, which is involved in progression of melanoma and HCC [164]. Also, extracellular acidification promotes invadopodia formation that enhances exosome secretion and cancer metastasis [128, 165]. Marwa, et al. showed that Pantoprazole which inhibits V-ATPases and increases extracellular pH could suppress exosome biogenesis and attenuate liver tumorigenesis [166].

### Lactate

Extracellular lactate produced by cancer cells promotes tumor-associated macrophages (TAMs) to secrete exosomes that contain HIF-1α-stabilizing long noncoding RNA, which are absorbed by breast cancer cells to enhance aerobic glycolysis and apoptotic resistance. And, knocking down Rab27 in TAMs inhibits exosome secretion and abolishes the effects of TAMs on cancer cells [167]. In addition, Yang et al. found that extracellular lactate promoted lactylation and exosomal release of HMGB1 from macrophages [168]. Notably, exosomal HMGB1 was proposed to promote cancer cell survival and immune evasion [169]. Thus, it is appropriate to investigate whether and how lactate has a more general function in exosome secretion and cancer progression.

### Signaling pathways

**Fig. 6:**
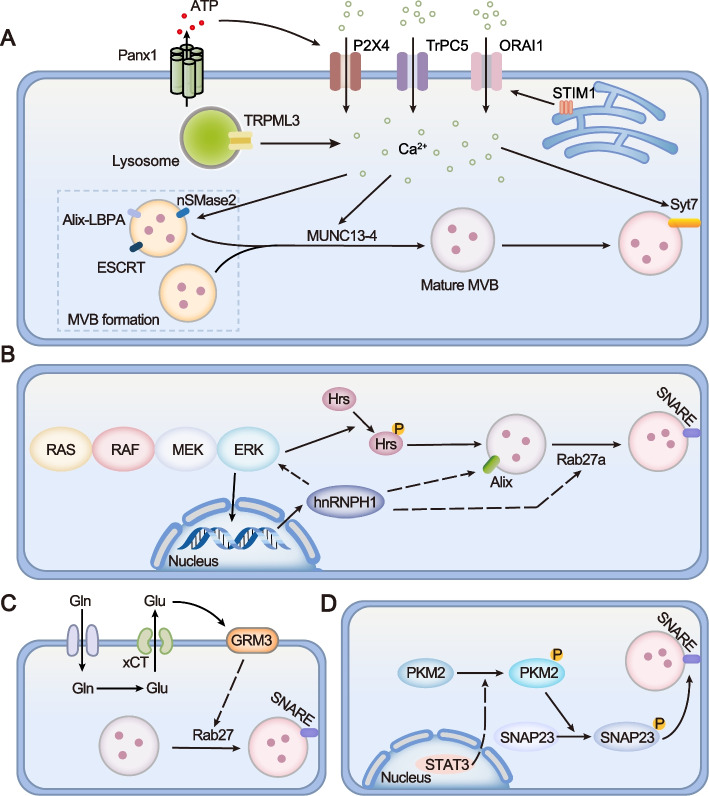
Aberrant activation of signaling pathways regulates exosome secretion in cancer. **A.** Increased Ca^2+^ in the cytosol that was caused by various stimuli contributes to the activity and function of multiple proteins mediating exosome generation, such as ESCRT, Alix-LBPA interaction, nSMase2, Munc13-4, and Syt7. **B.** RAS/RAF/MEK/ERK pathway signaling promotes the transcription of hnRNP H1, through which to facilitate exosome biogenesis by upregulating the expression of Alix and Rab27a. Moreover, there is a positive feedback loop between Ras signaling and hnRNP H1. In addition, ERK interacts with and phosphorylates Hrs, thereby promoting exosome secretion. **C.** Glutamine importer ASCT2 transfers extracellular glutamine (Gln) into the cell, where it can be converted into glutamate (Glu). Subsequently, intracellular glutamate is exported outside the cell by xCT. After binding to its receptor GRM3, GRM3 promotes Rab27-dependent exosome release. **D.** Activation of STAT3 promotes sequential phosphorylation of PKM2 and SNAP23, thereby accelerating the formation of the SNARE complex and exosome secretion

Aberrant activation of several signaling pathways exerts their functions in cancer progression by modulating the generation and composition of exosomes (Fig. 6).

### Ca^2+^ signaling

Dysregulation of Ca^2+^ signaling and altered levels of intracellular Ca^2+^ are involved in a plethora of processes in carcinogenesis, progression and chemoresistance of multiple cancer cell types [170]. Mounting evidence has shown that an overload of cytosolic Ca^2+^ due to either release from the intracellular Ca^2+^ store or influx from the extracellular milieu facilitates exosome biogenesis in both physiological and pathological conditions [97, 171] (Fig. 6A). For instance, TRPML3 is a transient receptor potential cation channel localized on lysosomes. Neutralized lysosomal pH caused by bacterial infection triggers Ca^2+^ release and subsequently stimulates the secretion of exosomes encasing bacteria in bladder epithelial cells [172]. Transient receptor potential channel 5 (TrpC5), a Ca^2+^-permeable cation channel, promotes exosome secretion, through which chemoresistance is transferred to recipient cells in breast cancer [173]. STIM1 senses decreased Ca^2+^ in endoplasmic reticulum and activates plasma membrane localized ORAI1, thereby inducing store-operated Ca^2+^ entry (SOCE). Panda et al. found that knocking down STIM/ORAI1 or decreasing Ca^2+^ levels using the SOCE inhibitor YM58483 could suppress exosome secretion in breast cancer cells [174]. In addition, Kim et al. reported that hepatitis C virus (HCV) infection stimulated Panx1-induced ATP release, which in turn activated purinergic receptor P2X4, thereby enhancing cytosolic Ca^2+^ and accelerating exosome secretion in human hepatoma cells [175]. Mechanistically, Ca^2+^ is vital for the activity and function of multiple proteins mediating exosome generation, such as ESCRT, Alix, nSMase2, Munc13-4, and Syt7 [98, 111, 175–177]. As described earlier, these proteins are involved in ILV formation, MVB maturation and MVB- plasma membrane fusion.

### Ras/Raf/MEK/ERK signaling pathway

RAS is one of the most frequently mutated oncogenes. Demory et al. reported that, in colorectal cancer (CRC) cells, mutant KRAS boosted the secretion of exosomes containing EGFR, SRC family kinases, and integrins, which promote the invasiveness of recipient cells [178]. Cha et al. reported that mutant KRAS expressed in CRC cells selectively promoted exosome secretion of miRNA-100, while wild-type KRAS accelerated exosome export of miRNA-10b, which is consistent with other report performed in the mouse pancreatic cancer model induced by mutant KRAS [140]. Moreover, this group found that inhibiting nSMase with GW4869 blocked miRNA-100 secretion only in mutant KRAS-expressing cells, but has no effects on miRNA-10b export in wild-type cells. These results further suggest that multiple routes might exist to sort distinct miRNAs into exosomes and that the nSMase2-ceramide dependent pathway is essential for mutant KRAS but not wild-type KRAS to exert its role in exosome secretion of miRNAs. Additionally, RAS/RAF/MEK/ERK pathway could promote the transcription of hnRNP H1 and the latter facilitates exosome biogenesis by upregulating the expression of Hrs, ALIX and Rab27a in prostate cancer cells [152]. Notably, hnRNP H1 intensifies the activation of Ras signaling by mediating the alternative splicing of A-Raf, implying that there is a positive feedback loop between Ras signaling and hnRNP H1 [152, 179] (Fig. 6B). Guan et al. found that activated ERK1/2, downstream of mutant KRAS, interacts with and phosphorylates Hrs, a crucial component of the ESCRT complex. Following the phosphorylation, Hrs binds to PD-L1 and promotes its exosome secretion in melanoma [180]. Conversely, Alix was proposed to interact with RNA-binding protein Ago2 and mediate exosomal sorting of Ago2 together with its binding miRNAs in human liver stem-like cells [76]. McKenzie and Cha groups reported that MEK-ERK signaling promoted phosphorylation of Ago2, inhibited its association with MVBs and suppressed exosomal secretion of Ago2 and its binding miRNAs in CRC cells [181]. Collectively, RAS signaling directly regulates the sorting of a variety of cargos into exosomes, although how mutant KRAS promotes specific miRNA secretion remains an open question. Of note, oncogenic KRAS can be detected in exosomes derived from colon cancer and pancreatic ductal adenocarcinoma cells, and oxidative stress could enhance its exosomal secretion through autophagy-dependent pathway [178]. Thus, it is conceivable that aberrant RAS signaling could be transferred to other cells through exosomes and further modify exosome biogenesis in recipient cells.

### Glutamine and glutamate

High glutamine consumption is a hallmark of cancer metabolism and plays a fundamental role in cancer progression [182]. Glutamine importers such as sodium-dependent neutral amino acid transporter type 2 (ASCT2) mediate the uptake of extracellular glutamine into the cell, where it can be converted into glutamate and other metabolites through glutaminolysis. Subsequently, intracellular glutamate is exported outside the cell by cystine/glutamate antiporter System Xc^−^. After binding to its receptor metabotropic glutamate receptor 3 (mGluR3, encoded by GRM3), extracellular glutamate activates mGluR3 to promote Rab27-dependent exosome release [12, 183] (Fig. 6C). For instance, Rabas et al. found that glutamine or glutamate-replete medium could enhance the secretion of CD63 positive and mitochondrial DNA containing exosomes. These exosomes facilitated the invasiveness of recipient cells in breast cancer, which could be inhibited by the mGluR3 antagonist LY341495 [12]. Fan et al. reported that glutamine depletion leaded to the switch of exosome subtypes from CD63-positive to Rab11-positive in human colorectal cancer cells. In particular, these Rab11-positive exosomes promote tumor growth and blood vessel formation in vitro and in vivo [99]. Nevertheless, how glutamate signaling activates the Rab27-dependent pathway and whether other mechanisms, except for Rab27, are involved in glutamate signaling mediated exosome biogenesis are underdetermined. In summary, exosomes can mediate metabolic reprogramming in cancer [7], and in turn, cancer metabolism can modulate the biogenesis and composition of exosomes.

### STAT3 pathway

STAT3 pathway is aberrantly activated in cancer and is a potential target for cancer therapy. Dorayappan et al. found that inhibiting STAT3 blocked exosome secretion by upregulating Rab27a and downregulating Rab7 [154]. Recently, STAT3 was proposed to be directly involved in exosome biogenesis. Mechanistically, activation of STAT3 promoted sequential phosphorylation of PKM2 and SNAP23, thereby accelerating the formation of the SNARE complex and exosome secretion (Fig. 6D). Thus, by regulating exosome biogenesis, the activation of STAT3/PKM2/SNAP23 pathway contributes to cancer cachexia development [184].

### GPCR signaling

GPCRs can not only be secreted into exosomes, but can also regulate exosome biogenesis. For instance, histamine H1 receptor (H1HR) is a GPCR that is activated by histamine. Activation of H1HR–Gα_q_–PKC pathway phosphorylates SNAP23 and promotes exosome production in HeLa cells [185]. In addition, endothelin receptor A (ETA), another GPCR, is aberrantly activated and contributes to malignant progression in various cancers. Im et al. showed that ETA enhanced the transcription of multiple genes, such as Rab27a, Rab5, and Vps4B, to promote exosome biogenesis in breast cancer [186].

### p53

Mutant p53 modulates the tumor microenvironment to support cancer invasiveness by controlling exosomal podocalyxin levels and increasing exosomal secretion of Hsp90α [74, 187]. Cooks et al. showed that mutant p53-expressing cancer cells selectively secreted exosomal miRNA-1246, which reprogrammed macrophages to be tumor supportive in colon cancer [188]. On the other hand, wild-type p53 upregulates the transcription of target genes CHMP4C, caveolin-1, and TSAP-6 to enhance exosome production[189]. In addition, acetylation of wild-type p53 by the BAG6/CBP/p300-acetylase complex promotes exosomal secretion of BAG6, which inhibits metastasis of melanoma cells [31]. Nevertheless, the precise mechanism by which mutant and wild-type p53 modulate exosome secretion is largely unknown.

### mTOR signaling

In addition to its function in autophagy, mTOR signaling is involved in exosome generation. Inhibiting mTORC1 signaling with rapamycin or asteltoxin stimulates the nuclear translocation of the transcription factor TFE3 and promotes the transcription of lysosome-associated genes Lamp1 and V-ATPase subunit d2. In this way, mTORC1 inactivation promotes degradation of MVBs and reduces exosome release in human prostate cancer PC3 cells and colon cancer HT29 cells [190]. Conversely, Zou et al. showed that inactivation of mTORC1 with rapamycin accelerated exosome release in mouse embryo fibroblasts [191]. Additionally, mTORC1 regulates glutamine metabolism and promotes PD-L1 expression and its exosomal secretion in hepatocellular carcinoma [192]. Thus, mTOR signaling plays distinct roles in exosome biogenesis according to different cell types or exosomal cargo.

### FGF2 signaling

FGF2 is a proangiogenic factor and plays a vital role in cancer angiogenesis and cell survival [193]. Nathalie et al. found that FGF2 was released into the extracellular environment primarily within exosome/small vesicles via its interaction with FGFR1. Activation of FGF2/FGFR1 signaling in bone marrow stromal cells promoted the secretion of FGF2/ FGFR1-laden exosomes, which were internalized by leukemia cells and contributed to their resistance to tyrosine kinase inhibitors [194].

### microRNAs

By negatively regulating the expression of target genes, several microRNAs are proposed to function in exosome biogenesis (Table 2). For example, YKT6 is target of miR-134 and miR-135b, and, overexpression of miR-134 and miR-135b reduces exosome release in non-small cell lung cancer [195]. By directly targeting SNAP23, let-7a regulates exosome secretion and functions as a tumor suppressor in colorectal cancer [196]. By performing high-throughput miRNA-based screening in prostate cancer cells, Urabe et al. found that miR-26a was involved in exosome release by targeting SHC4, PFDN4, and CHORDC1 [197]; yet the precise function of these target genes in exosome generation is unknown.

**Table 2:** Factors that regulate exosome biogenesis

	Genes	Roles on exosome biogenesis	Targets	Cancer cell types	Refs
microRNAs	Let-7a	Inhibit	SNAP23	CRC	[196]
	miR-134 and miR -135b	Inhibit	YKT6	NSCLC	[195]
LncRNAs	LncRNA-APC1	Inhibit	Rab5b	CRC	[198]
	LINC00511	Enhance	RAB27B, VAMP7-SNAP23 complex	HCC	[127]
	PVT1	Enhance	YKT6, RAB7, and VAMP3	Pancreatic cancer	[199]
	HOTAIR	Enhance	Rab35, SNAP23	HCC	[135]
PTMs	Phosphorylation	Enhance	Syntenin, SNAP23, Hrs	Breast cancer, melanoma	[23, 180, 184]
		Inhibit	Ago2	Colon cancer	[181]
	SUMOylation	Enhance	hnRNPA1, hnRNPA2B1	Bladder cancer	[84, 85]
	O-GlcNAclation	Inhibit	SNAP-23	Ovarian and Breast cancer	[141]
	Neddylation	Inhibit	Coro1a	Colon cancer	[102]

*HCC* Hepatocellular carcinoma, *NSCLC* Non-small cell lung cancer, *CRC* Colorectal cancer

### lncRNAs

lncRNAs have important functions in the transport of MVBs by regulating the transcription or localization of target proteins (Table 2). For example, tumor suppresser lncRNA-APC1 interacts with Rab5b mRNA and reduces mRNA stability. By down regulating Rab5b, lncRNA-APC1 inhibits the production of tumor-promoting exosomes and progression of colorectal carcinoma [198]. LncRNA HOX transcript antisense RNA (HOTAIR) acting as an oncogene promotes exosome secretion in hepatocellular carcinoma. HOTAIR regulates expression of Rab35 and promotes colocalization of VAMP3 and SNAP23 to facilitate MVB-plasma membrane fusion [135]. Peng et al. found that, by regulating the colocalization of VAMP7 and SNAP23, LINC00511 induced invadopodia formation and promoted MVB fusion with the plasma membrane and exosome secretion in hepatocellular carcinoma [127]. Sun et al. reported that lncRNA plasmacytoma variant translocation 1 (PVT1) promoted exosome secretion from pancreatic cancer cells by controlling the expression and localization of Rab7 and regulating the colocalization of YKT6 and VAMP3 [199].

### Post transcriptional modifications

Dysregulation of posttranscriptional modifications (PTMs) is important in the progression of various cancers. In addition to phosphorylation of Syntenin, SNAP23 and Ago2, which control exosome biogenesis in cancers, as described earlier, other PTMs regulate the quantity and composition of exosomes by modulating elements of the machinery of exosome biogenesis (Table 2). For example, downregulation of O-GlcNAclation transferase (OGT) reduces O-GlcNAc modification of SNAP-23, thereby promoting the formation of SNARE complex composed of SNAP-23, VAMP8, and Syntaxin-4 and eventually promoting exosome biogenesis in ovarian cancer cells [141]. In addition, GlcNAc modification can influence the function of exosomes in other cancers [200]. Fei et al. showed that inhibition of neddylation of Coro1a enhanced exosome secretion and promoted tumor progression in HeLa and MC38 mouse colon cancer cells [102]. Villarroya-Beltri et al. reported that SUMOylation of hnRNPA2B1 promoted exosomal sorting of its binding miRNAs [201]. Chen et al. showed that hnRNPA1 was SUMOylated by SUMO-2, which contributed to exosomal secretion of lncRNA ELNAT1 and ultimately promoted the lymph node metastasis of bladder cancer [85]. Additionally, ISGylation of Tsg101 mediated by ISG15 promotes its degradation and inhibits exosome generation in HEK293T and Jurkat T cells [103]. Giovannone et al. reported that mono-ubiquitination of syntaxin-3 promoted its function in exosome biogenesis in MDCK cells [202]. Also, investigators have proposed that Flotillins are modified by palmitoylation and that palmitoylation of Dsg2 promotes secretion of exosomes with mitogenic content [47, 203].

### Therapeutic implications

As described earlier, various mechanisms mediating exosome biogenesis are dysregulated in cancer progression. Thus, targeting exosome biogenesis is a promising strategy for cancer therapy. Importantly, pharmacological targeting of exosome generation has been shown to be beneficial in the treatment of cancer (Table 3).

**Table 3:** Potential molecules that regulate exosome biogenesis in cancer

Inhibitor	Tagetes	Pathways	Cancer cell types	Refs
GW4869	nSMase2	nSMase2-ceremide pathway	Breast cancer, prostate cancer, melanoma, glioma, and myeloma	[68, 69, 204, 205]
SyntOFF	Syntenin	Syndecan-Syntenin-Alix	Breast cancer	[206]
Halopemide	PLD2	Syndecan-Syntenin-Alix	Prostate cancer	[207]
CAY10594	PLD2	Syndecan-Syntenin-Alix	Breast cancer	[71, 208]
Dendrogenin A	LXRβ	Rab27a, LBPA	Melanoma, breast cancer	[209]
Sulfasalazine	System Xc^−^	Glutamate-GRM3-Rab27a	Multiple myeloma	[183]
CPPG	GRM3	Rab27a and Alix	Multiple myeloma	[183]
LY341495	GRM3	Rab27a	Breast cancer	[12]
BHMPS	Rab27a	Rab27a- Slp4	Breast cancer	[109]
R491	Vimentin	-	Lung cancer, pancreatic cancer, glioma, HCC	[210]
Sulfisoxazole	Endothelin receptor A	Multiple process	Breast cancer	[211]
Macitentan	Endothelin receptor A	Multiple process	Breast, colon and long cancer	[212]
A740003 and AZ10606120	P2X7R	-	Melanoma	[213]
Manumycin A	Farnesyltransferase	Ras/Raf/ERK1/2 signaling	prostate cancer	[152]
Tipifarnib	Farnesyltransferase	Ras/Raf/ERK1/2 signaling	Rhabdomyosarcoma, prostate cancer	[214]
SCH772984	ERK1/2	Ras/Raf/ERK signaling	Melanoma	[180]
U0126	MEK1/2	Ras/Raf/ERK signaling	Prostate cancer	[214]
Asteltoxin	Mitochondrial ATP synthase	AMPK/mTOR	HeLa, colon and prostate cancer	[190]
Pantoprazole	V-ATPase	Extracellular pH	Liver cancer	[166]
LDN-57444	DUB	-	OSCC, NP	[215]
PD173074, BGJ-398	FGFR1	FGF2/FGFR1 signaling	Leukemia	[194]

*HCC* Hepatocellular carcinoma, *OSCC* Oral squamous cell carcinoma, *NP* Nasopharyngeal

### Targeting nSMase2

The nSMase2-ceremide pathway is vital for ESCRT-independent exosome biogenesis. GW4869 is the first reported and noncompetitive nSMase2 inhibitor [216]. It is widely used as a tool to determine the sorting mechanism and function of exosomal cargos. For instance, GW4869 inhibits exosome secretion of programmed death-ligand 1 (PD-L1) from cancers such as breast cancer, prostate cancer, and melanoma, thereby sensitizing anti-PD-L1 therapy [68, 69, 204]. Cancer-associated fibroblasts secrete exosomes that contain miR-21, miR-181a, miR-221, miR-222, and miR-92a via the nSMase2-ceremide dependent pathway. GW4869 could inhibit the secretion of these exosomes to restrain their promoting function in the proliferation and chemoresistance of pancreatic ductal adenocarcinoma. In addition, the tumor-suppressor miR-375, which targets the mRNA of connective tissue growth factor (CTGF), can be sorted into exosomes from glioma cells. GW4869 inhibits miR-375 release, down-regulates the activity of the CTGF-EGFR pathway in glioma cells and impairs the malignant progression of glioma [205]. Therefore, by reducing exosome sorting of target cargos, GW4869 might be an effective anti-tumor agent. However, GW4869 is not suitable for further clinical development because of its low solubility and inhibitory potency[216, 217]. Several other specific nSMase2 inhibitors with potential for clinical application have been exploited, such as 2,6-dimethoxy-4-(5-phenyl-4-thiophen-2-yl-1H-imidazol-2-yl)-phenol (DPTIP) and phenyl(R)-(1-(3-(3,4‐dimethoxyphenyl)‐2,6-dimethylimidazo[1,2‐b]pyridazin-8-yl)pyrrolidin-3-yl)-carbamate (PDDC) [217, 218]. But their activities in exosome biogenesis and cancer progression need clarification.

Of note, ceramide is widely acknowledged as a lipid tumor suppressor. By accelerating ceramide production, nSMase2 can induce differentiation and apoptosis while inhibiting the proliferation and chemoresistance of a variety of cancers [219]. Conversely, by mediating exosome release of different cargos, the nSMase2-ceramide pathway promotes the progression of many cancers [37, 69, 220]. Thus, the nSMase2-ceramide pathway plays dual roles in the progression of cancer. Additionally, nSMase2 is localized mainly to the Golgi and plasma membrane [221]. On the plasma membrane, following activation of TNF, nSMase2 interacts with embryonic ectodermal development (EED) and is recruited to the TNF-R1/FAN/RACK1 complex, which regulates the cell cycle and apoptosis [222]. Stress stimuli (such as infection and radiation), activated receptors (e.g., CD40, P2X7) or phosphatidylserine stimulate nSMase activity and contribute to the formation of ceramide-enriched lipid rafts on the plasma membrane [40, 41, 223]. However, little is known about how the nSMase2-ceramide pathway is directly regulated or activated on the limiting membrane of MVBs. Leidal et al. proposed that exosome generation mediated by the LC3-conjugation machinery required both nSMase2 and its activator FAN [39]. However, Philipp et al. did not find a direct interaction between FAN and nSMase2 [222], and FAN did not affect the activity of nSMase2 without binding to TNF-R1 on the plasma membrane [38]. Although TNF-R1 can be released into exosomes[224], the exact mechanism that regulates the nSMase2-ceramide pathway on the limiting membrane of MVBs is still obscure. STARD11 and DEGS1 mediate ceramide trafficking to or production on MVBs, separately, but robust evidence remains to be established, and the functions of these proteins in cancer remain unclear [39, 225, 226]. Therefore, the different functions of nSMase2 might depend on its particular cellular location. Elucidating the exact molecular mechanism that regulates the translocation and activation of nSMase2 on MVBs may be helpful for recognizing specific therapeutic targets for cancer.

### Targeting the Syndecan-Syntenin-Alix pathway

Syntenin acts as an oncogene and plays a central role in exosome biogenesis mediated by the Syndecan-Syntenin-Alix pathway, and the PDZ domain of Syntenin is vital for its activity [145, 227]. Leblanc et al. screened a PDZ-targeted compound library and, with structural optimization, developed a novel Syntenin inhibitor, SyntOFF [206]. SyntOFF reduced the proliferation and metastasis of breast cancer and decreased the secretion of exosomes that contained c-Src and EpCAM which are important for cancer progression. PLD2 is a regulator of the Syndecan-Syntenin-Alix pathway for exosome biogenesis and acts as an oncogene in several cancer types. Halopemide is a non-specific PLD2 inhibitor that can reduce prostate cancer cell-derived exosome secretion and incapacitate these exosomes to stimulate the proliferation and mineralization of osteoblasts. Because osteosclerotic behavior is a feature of the bone metastatic niche, it was proposed that targeting PLD2 could prevent or retard bone metastasis of prostate cancer [207]. In addition, CAY10594, an optimized analog of halopemide, is a selective PLD2 inhibitor. Treatment of breast cancer cells with CAY10594 decreased ILV formation and exosome secretion mediated by the Syndecan-Syntenin-Alix pathway [71]. Interestingly, CAY10594 was reported to decrease the infiltration of tumor-associated macrophages and neutrophils and suppress the proliferation and metastasis of breast cancer [208].

### Targeting cholesterol metabolism pathway

Cholesterol is required for ILV formation and exosome secretion in several cancers. Statins are inhibitors of HMG CoA reductase and are widely used in the clinical treatment of high cholesterol. Kulshreshtha et al. reported that simvastatin reduced exosome secretion from several macrophage and epithelial cells [228]. In contrast, atorvastatin did not affect the morphology and quantity of exosomes secreted from mesenchymal stem cells. However, Huang et al. found that atorvastatin increased lncRNA H19 secretion in mesenchymal stem cell-derived exosomes which promoted tube formation of HUVEC and displayed cardioprotective effects during acute myocardial infarction [229]. Kuo et al. found that simvastatin promoted decorin but inhibited periostin secretion in exosomes derived from cardiomyocytes, thereby reducing cardiac fibrosis [230]. Considering that statins inhibit tumor progression in multiple cancers, it will be interesting to determine whether statins exert their anticancer properties by regulating exosome secretion.

Dendrogenin A (DDA) is a natural cholesterol-derived metabolite and acts as the ligand of nuclear liver X receptors (LXR), a transcription factor that functions in lipid metabolism and immune response. An increasing number of studies performed in vitro and in vivo have indicated that DDA is a tumor suppressor and promotes differentiation and cell death in various cancers, such as melanoma, breast cancer, and acute myeloid leukemia [209]. Record et al. showed that compound DDA functioned through LXRβ and promoted the production of bis(monoacylglycero)phosphate (BMP, also known as LBPA) and the expression of Rab27a in melanoma and breast adenocarcinoma cells. Consequently, DDA treatment promotes the secretion of exosomes that contribute to the maturation of human dendritic cells and subsequently the activation of T lymphocytes [231]. Thus, DDA or targeting LXR provides a novel avenue for antitumor immunity in an exosome-dependent manner.

### Targeting the glutamate/GRM3/Rab27a pathway

The xCT (encoded by SLC7A11) is a functional subunit of glutamate antiporter system Xc^−^ and is overexpressed in many cancers [232]. Recently, it was reported that system Xc^−^ mediated glutamate export enhanced exosome secretion in multiple myeloma and bone marrow stromal cells by upregulating the expression of Rab27a, TSG101, Alix, and VAMP7, thereby contributing to bortezomib resistance in multiple myeloma. Targeting system Xc^−^ with the inhibitor sulfasalazine (SASP) or targeting GRM3 with the antagonist (RS)-alpha-cyclopropyl-4-phosphonophenylglycine (CPPG) reduced exosome secretion and countered bortezomib resistance in multiple myeloma [183]. However, although SASP treatment causes reactive oxygen species (ROS)-related apoptosis and suppresses cell growth in melanoma, it promotes the expression and exosomal release of PD-L1, leading to polarization of M2 macrophages and tolerance to anti-PD-1/PD-L1 therapy [233]. Thus, SASP is not suitable for combined use with anti-PD-1/PD-L1 therapy, at least in melanoma. Notably, Rab27a is vital for exosomal release of PD-L1 in melanoma [69], but whether SASP regulates the expression of Rab27a in melanoma is unknown. Notably, (E)-N-benzyl-6-(2-(3, 4-dihydroxybenzylidene) hydrazinyl)-N-methylpyridine-3-sulfonamide (BHMPS) is a novel Rab27a inhibitor that specifically disrupts the association of Rab27a with its effector Slp4. It has been shown that BHMPS blocks exosome secretion and attenuates the invasion and migration of breast cancer [109]. Moreover, sulfisoxazole and macitentan are FDA-approved antagonists of endothelin receptor A (ETA) which regulates the expression of a series of genes associated with exosome biogenesis, such as Rab27a and Vps4B. Both of these drugs could reduce the exosomal secretion of PD-L1 and improve the response to anti-PD-1/PD-L1 therapy in cancer, such as breast, colon, and lung cancer [211, 212].

### Targeting Ras signaling pathway

The Ras signaling pathway is frequently mutated and activated in various cancers. The farnesylation of Ras plays an important role in its membrane localization and signal transduction [234]. Manumycin A, a natural antibiotic, is a selective inhibitor of farnesyltransferase (FTase). By inhibiting farnesylation of Ras, Manumycin A suppresses Ras/Raf/ERK1/2 signaling and reduces exosome generation in castration-resistant prostate cancer [152]. Consistently, tipifarnib, another selective inhibitor of Ras farnesylation, inhibits exosome biogenesis in prostate cancer cells by decreasing Ras/Raf/ERK signaling [214]. Moreover, the MEK1/2 inhibitor U0126 impairs exosome generation in prostate cancer [214]. Guan et al. showed that ERK inhibitor SCH772984 suppresses the phosphorylation of HRS and blocks the exosomal secretion of PD-L1 in melanoma cells. Consequently, SCH772984 promotes the migration of CD8 + T cells into tumors and enhances the efficacy of cancer immunotherapy [180]. In summary, a link between Ras/Raf/ERK signaling and exosome generation in cancer has been well established. Targeting exosome biogenesis might be crucial for Ras signaling inhibitors to exert their anticancer effects.

### Others

Several other inhibitors have also been developed to block exosome biogenesis in cancer. For instance, targeting FGFR with the inhibitors PD173074 and BGJ398 (infigratinib) suppresses the exosomal secretion of FGF2 and protects leukemia cells from tyrosine kinase inhibitors [194]. Notably, infigratinib has been approved by the FDA for the treatment of cholangiocarcinoma [235]. P2X7R is an ATP-gated ion channel, and its activation accelerates exosome biogenesis [236]. Targeting P2X7R with selective inhibitor A740003 or AZ10606120 suppresses exosomal secretion of migration-associated miRNAs and inhibits metastasis of melanoma cells [213]. BG45, an HDAC3-selective inhibitor, downregulates TSG101 and reduces the secretion of exosomes loaded with tumor-promoting miRNAs in multiple myeloma [237]. LDN-57444, an inhibitor of ubiquitin C-terminal hydrolase L1 (UCH-L1), decreases the release of pro-metastatic protein-laden exosomes in human oral squamous carcinoma cells [215]. The small-molecule compound R491 binds to vimentin, impairs vimentin-mediated MVBs transport and exosome release, and thus inhibits the motility of various cancers [210].

## Conclusion

Much progress has been made in understanding the important roles of exosomes in cancer progression, but the precise mechanism mediating exosome biogenesis, especially the mechanism leading to the heterogeneity of exosomes remains relatively understudied. Aside from the heterogeneous nature of exosomes, there are several other challenges. Exosome biogenesis consists of four steps, each of which is regulated by multiple mechanisms. How do these distinct mechanisms cooperate in a fine-tuned manner? In addition, the exact mechanism of sorting most cargos to exosomes is far from clear. For example, how exactly are most RNAs and RNA-binding proteins sorted into MVBs? Third, recent studies have uncovered several different subpopulations of MVBs; are there any more subfamilies of MVBs? What is the mechanism of their generation? Of note, considering that the mechanisms of exosome biogenesis are highly variable depending on the cargo, cell type, and context, caution should be taken in making conclusions about the particular mechanism observed in one study.

Cancer cells employ a variety of strategies to hijack mechanisms of exosome biogenesis to promote their progression. Therefore, delineating the machinery has great significance for therapeutic implications. The International Society for Extracellular Vesicles (ISEV) has highlighted the perspective of extracellular vesicles as theranostics [238]. To date, many compounds have been identified to target exosome biogenesis, and preclinical studies have revealed their therapeutic potential. Nevertheless, clinical trials are still lacking. Further study should evaluate their clinical value and side effects. Moreover, given the functions of exosomes in tumor microenvironment, the combined use of exosome-targeting compounds with other antitumor drugs, such as anti-PD-1/PD-L1 or chemotherapeutics, might be a potential strategy for cancer therapy.

A better understanding of exosome biogenesis is of great significance for other clinical applications of exosomes. For example, as the composition of exosomes differs between healthy individuals and cancer patients, exosomes are proposed biomarkers for the diagnosis or evaluation of the progression of cancer [239, 240]. However, extracellular vesicles are highly heterogeneous both in their physical characteristics and molecular composition. This heterogeneity poses significant challenges for isolation strategies and analysis of the composition and function of exosomes. Thus, elucidating the mechanisms of exosome biogenesis could promote a better understanding of the composition of exosomes and identify better diagnostic markers in cancer. In addition, exosomes can be used as a drug delivery system for cancer therapy [241, 242]. To date, multiple cell types, such as neutrophils, dendritic cells, mesenchymal stem cells, HEK-293 T cells, and even tumor cells, have been used to obtain the desired exosomes/EVs [243, 244]. However, the low yield of exosomes is a major obstacle for their therapeutic implementation. Additionally, the heterogeneity would yield side effects of engineered exosomes/EVs. Thus, modulating the machinery of exosome biogenesis and precisely increasing the production of exosomes/EVs with required functions in a suitable cell type will have great value in clinical applications.

## Data Availability

Not applicable.

## Abbreviations

MVBs: Multivesicular bodies
LM: Limiting membrane
ILVs: Intralumenal vesicles
TGN: Trans-golgi network
ER: Endoplasmic reticulum
PM: Plasma membrane
ESCRT: Endosomal sorting complex required for transport
PI3P: Phosphatidylinositol (3)-phosphate
FGFR: Fibroblast growth factor receptor
KRS: ARS lysyl-tRNA synthetase
HPA: Heparanase
ARF6: ADP ribosylation factor 6
GPCRs: G protein-coupled receptors
PLD2: Phospholipase D2
PA: Phosphatidic acid
LBPA: Lysobisphosphatidic acid
nSMase2: The neutral sphingomyelinase 2
PLP: Proteolipid protein
LLPS: Liquid–liquid phase separation
HNSCC: Head and neck squamous cell carcinoma
HCC: Hepatocellular carcinoma cells
TME: Tumor microenvironment
TSPN6: Tetraspanin-6
miRNAs: Micrornas
lncRNAs: Long non-coding RNAs
